# Supplementary figures and images for: A hierarchical multiscale model of forward and backward alpha-band traveling waves in the visual system
Source: PLoS Comput Biol. 2025 Aug 11;21(8):e1013294. doi: 10.1371/journal.pcbi.1013294 (PMC12360655; doi:10.1371/journal.pcbi.1013294)

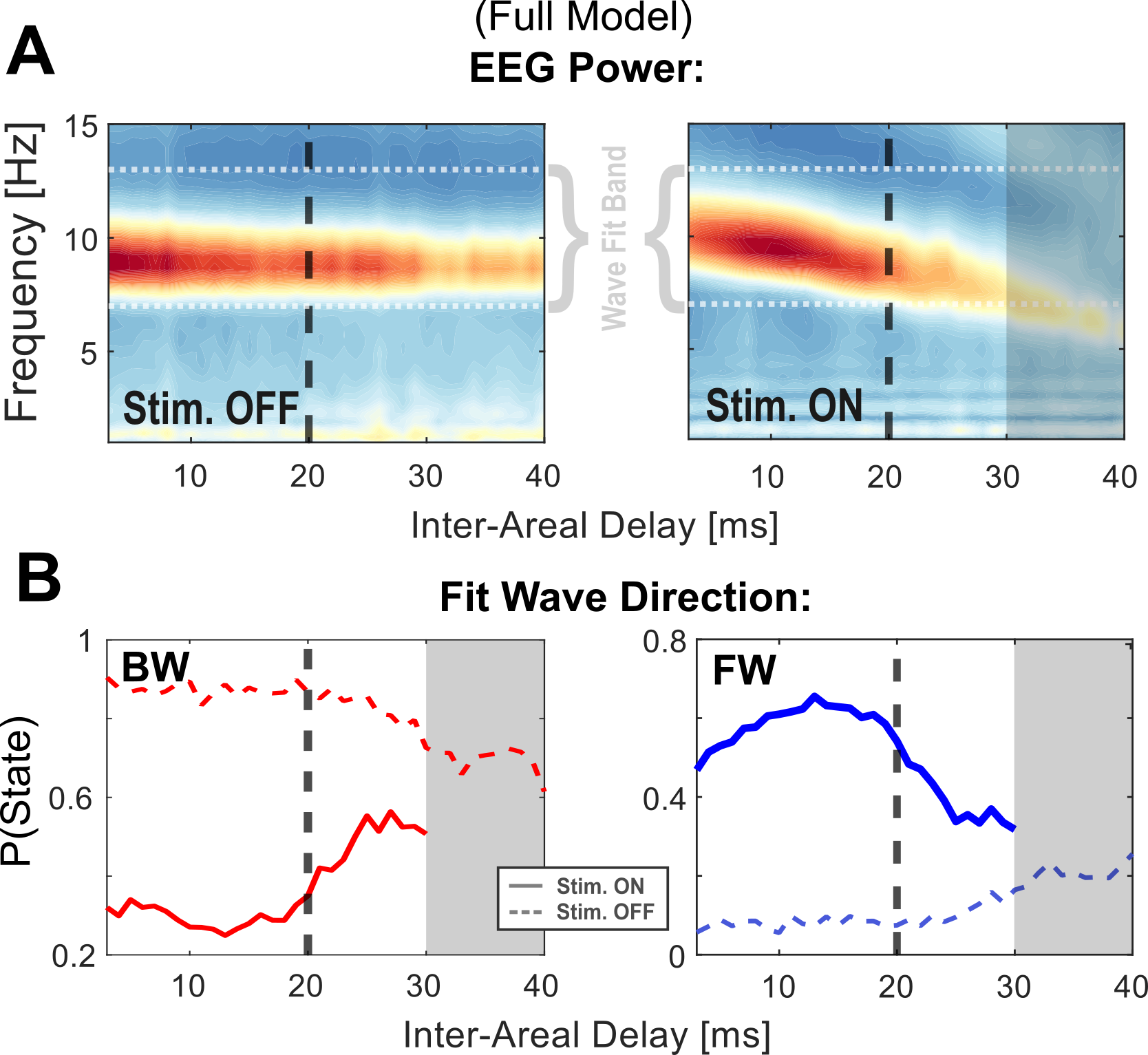

Supplement: S1 Fig — Data correspond to the full model with rhythmic IGIB activity and IGIB - IGIB feedback intact (compare Fig 3c, left panels). In this configuration, the network enters a diverging regime for delays > approx. 20 ms (dashed line), in which FW- and BW-pathways differ in their temporal frequencies. A: Mean power spectra (averaged between electrodes on the mid-line) during stimulus On and Off periods as a function of inter-areal delay. The temporal frequency of the scalp-level activity remains constant independent of delay in the stimulus Off period (left panel), but decreases along with the SGX frequency with increasing delay (right panel, cf. Fig 3c and 3d). In this state, the activity beings to move out of the frequency band used for the wave fit (alpha, 7–13 Hz) at approx. 30 ms delay (shaded area), therefore this range of delays is excluded in the fitting procedure below. B: Probabilites of FW and BW wave states as a function of delay, during stimulus On and Off periods. Simulations were run with an intermediate stimulus amplitude (I = 1.2 nA) between the reversal threshold and the upper asymptote to ensure dynamic range in the FW-state. The results show that the wave direction is stable within the non-diverging range of delays (up to 20 ms) while for higher delays, the FW state probability steadily decreases. (TIFF) [file pcbi.1013294.s002.tiff]

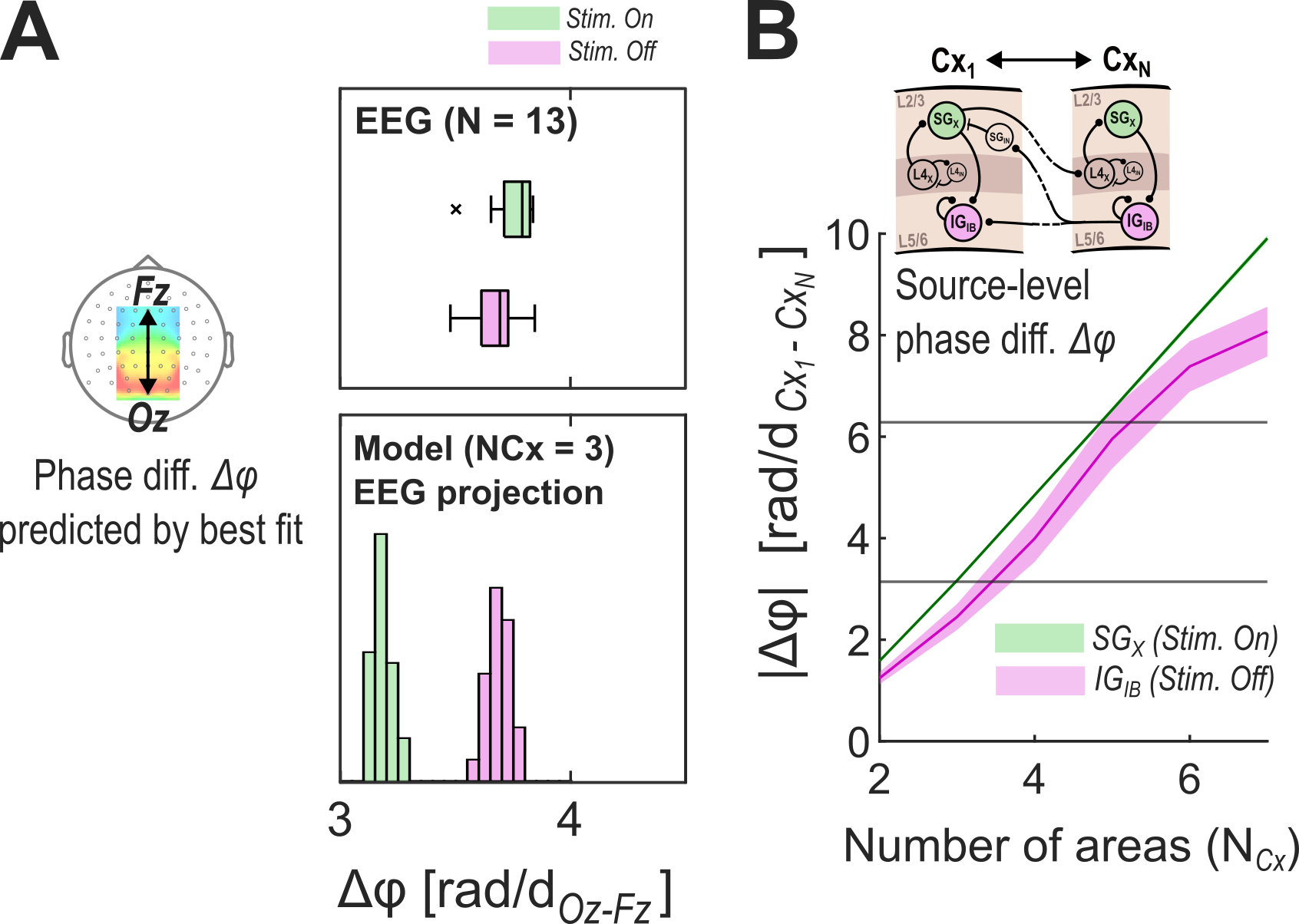

Supplement: S2 Fig — A: Spatial frequencies of planar wave fits, normalized to the phase difference predicted between Oz and Fz electrodes. Top panel shows boxplots of averages across participants in the real EEG data [14], bottom panel shows histograms of trial-averages obtained across 50 model simulations, using the same stimulus duration as the EEG experiment. The model EEG projection reproduces the empirical values for the BW state (stimulus OFF), but slightly overestimates the velocity of FW waves during stimulus ON periods. This effect is likely explained by our non-realistic EEG projection, which includes stronger transient activity at lower areas during the FW state (largely driven by sources in Layer 4; cf. the source level activity shown in Fig 1b, panel 4), and does not affect the direction of the fit (FW/BW). B: Source-level propagation velocities for models with different numbers of areas, normalized to the absolute phase difference covered by the entire hierarchy (Cx1 to CxN). Each model was run 50 times using the same stimulus configuration as in A, values shown are means + /- 1SD across model runs. The comparison between SGX and IGIB phase differences (respectively, during stimulus ON and OFF periods) shows that FW wave propagation is highly spatially consistent, while the spontaneous BW propagation velocity is more variable (cf. again Fig 1b, panels 4 and 5). Note that the linear relationship between number of modeled areas and total phase difference only holds under the simplifying assumption that all areas have the same neural time-constants. (TIFF) [file pcbi.1013294.s003.tiff]
